# Quantum Chemical Analysis of the Corrosion Inhibition Potential by Aliphatic Amines

**DOI:** 10.3390/ma14206197

**Published:** 2021-10-19

**Authors:** Szymon Malinowski, Michał Wróbel, Agnieszka Woszuk

**Affiliations:** Department of Building Materials Engineering and Geoengineering, Faculty of Civil Engineering and Architecture, Lublin University of Technology, Nadbystrzycka 40, 20-618 Lublin, Poland; m.wrobel@pollub.pl (M.W.); a.woszuk@pollub.pl (A.W.)

**Keywords:** aliphatic amine, corrosion inhibitor, Density Functional Theory (DFT), HSAB theory, corrosion protection efficiency

## Abstract

Destructive corrosion processes lead to the loss of primary mechanical properties of metal construction materials, which generates additional costs during their maintenance connected with repairs and protection. The effectiveness of corrosion inhibitors can be determined by using many methods, in particular quantum chemical modeling. The subject of the theoretical analyses presented in this work involves the anticorrosion properties of amines with various chemical structures. Evaluation of the corrosion inhibition properties of selected amines was performed on the basis of the HOMO–LUMO energy gap, dipole moment (µ), electronegativity (χ) determined as a result of the energy of the highest occupied molecular orbital (HOMO) and the energy of the lowest unoccupied molecular orbital (LUMO). Moreover, the HSAB (Hard and Soft Acids and Bases) theory was used to explain the reactivity of the analyzed amines, while the Mulliken population analysis was used to determine their electrostatic interactions with the surface of protected metal. The obtained results indicate that the protonation reaction of aliphatic amines leads to a change in the nature of the formation of a coordination bond with the surface of the protected metal. In turn, the quantum chemical calculations showed that the protonation reaction of aliphatic amines leads to a decrease in their corrosion inhibition efficiency. Most of the analyzed parameters indicated that tertiary amines are characterized by the highest corrosion inhibition efficiency.

## 1. Introduction

The main safety risks of metal building structures are the corrosion processes resulting in the transformation of the material into a more stable chemical form, e.g., oxide, hydroxide or sulfide, as a consequence of interaction with the environment. Various techniques are used to protect structural steel against the negative effects of corrosion, including protective coatings or corrosion inhibitors [1]. The latter are the substances added in low concentrations to the corrosion system in order to delay/reduce the intensity of the corrosion process due to adsorption of inhibitor molecules on the metal surface and formation of a protective layer preventing the access of aggressive ions to the surface of the protected metal [2]. Among many known corrosion inhibitors, amines and their derivatives [3,4,5], with diversified molecular structure, have different properties in terms of the efficiency of corrosion inhibition. Due to the destructive influence of corrosion processes on the mechanical properties of metal structures and consequently economic and safety factors of their application, one of the trends of the modern science and industry is the development of new types of corrosion inhibitors [6,7].

Amines are organic derivatives of ammonia in which one or more hydrogen atoms were replaced by hydrocarbon chains with different structures and numbers of carbon atoms. The number of hydrocarbon chains attached to the N atom indicates their subcategory. Primary, secondary and tertiary amines have one, two or three hydrocarbon chains attached to the N atom and are characterized by the existence of the -NH_2_, -NHR and -NR_1_R_2_ groups. As it was shown in previous studies, the protective effect of amines increases along with the number of hydrocarbon chains, which is related to the molecular charge of the N atom [8]. The factor determining the effectiveness of their anti-corrosion properties is the strength (durability) of the created amine–metal coordination bond and the solubility of the amine. On the other hand, the strength of the amine–metal interaction depends on the electron density around the nitrogen atom of a particular amine and their ability to form a coordination bond. Amines can be used as corrosion inhibitors, both in aqueous acid solutions as well as volatile inhibitors [3,5,9]. The basis of anti-corrosion properties of amine compounds is their adsorption on the surface of the protected metal, leading to the formation of a hydrophobic film, which significantly reduces the access of both water and aggressive ions, simultaneously delaying the anodic electrochemical corrosion processes of the metal [10]. The main disadvantages of using amine corrosion inhibitors are their toxicity, irritating odor and short effect. The selective action of amines in corrosion protection of metal structures is also problematic. The design of corrosion inhibitors requires the use of a specific amine for a particular metal. For example, some amines can protect steel, while promoting corrosion of other metals such as copper or bronze. Owing to the newly synthesized, optimized amine compounds, the negative properties of inhibitors can be avoided or at least significantly reduced without loss of the corrosion protection properties. In spite of many disadvantages, these compounds are widely used in the corrosion protection of construction materials, and therefore they are subject of many research and development studies. As indicated in the scientific literature, among a wide range of amino compounds, amino acids have been used as corrosion inhibitors. For example, tyrosine, methionine [11] alanine, cysteine and S-methyl cysteine [12] in iron protection, l-cysteine on bronze [13] and aluminum alloy [14], methionine and proline on carbon steel [15], tricine on zinc [16], glycine, alanine valine and tyrosine on copper [17] or proline on tin [18]. Moreover, anticorrosive properties have also been observed for many derivatives of amine compounds i.e., epoxy-amine [19] or THAM [20]. Farahati et al. used 4-(pyridin-3-yl) thiazol-2-amine for corrosion inhibition of copper in hydrochloric acid with theoretical and experimental methods, where MD simulations confirmed the results obtained by using conventional methods [21]. *N*,*N*′-bis(1-phenylethanol) ethylenediamine [22], diethylenetriamine I, triethylenetetramine II, pentaethylenehexamine III [23], 2,2′-(ethylenedioxy)diethylamine [24], 1,8-diaminooctane, tetraethylenepentamine [25], 4-amino-1-propyl-piperidine [26], methyldiethanolamine (MDEA), 2-amino-2-methyl-1-propanol (AMP), 1-(2-aminoethyl)piperazine (AEPZ) [27], N-(5-nitro-2-hydroxybenzylidene)pyridine-4-amine [28], triethylenetetramine (TETA) and 2-(2-aminoethylamino) ethanol (AEAE) [29] were also used in corrosion inhibition.

The corrosion process of metals can be studied by weight loss, electrochemical measurements, hydrogen evolution measurements, the thermo-metric method and microscopy techniques, etc. In recent years, there has been a significant increase in interest of computational chemistry method for studying the corrosion protection properties of organic compounds. Many researchers point to quantum-chemical methods as the most relevant tools in the analysis of the performance of metal corrosion inhibitors. Moreover, theoretical methods do not require time-consuming experimental investigations, so they are a convenient way of determining the inhibitor-metal interactions. The use of Molecular Dynamic (MD) simulation with periodic boundary condition was used to calculate the adsorption energy and to identify the adsorption configuration of several amino acids on metal surfaces [1]. Theoretical investigations provide the atomic level as well as the molecular level insights in the field of the corrosion inhibition chemistry [4]. Moreover, due to economic considerations, theoretical methods found a special place in the study of the anti-corrosion properties of organic compounds including amine compounds. Boughoues et al. [30] theoretically analyzed the anticorrosive properties of four derivatives of amine compounds using DFT and MD methods and determined their mechanism of inhibition, finding direct correlations with the results obtained using electrochemical methods. The experimental work carried out by Shihab and Al-Doori confirmed the physisorption effect of organic compounds during corrosion protection, and the theoretical models developed provided useful information to explain the interaction between the metal surface and selected inhibitors at the atomic level [31]. Khadom studied the possibility of corrosion inhibition of copper alloy by using phenylenediamine (PDA), tetraethylenepentamine (TEPA), diethylenetriamine (DETA) and ethylenediamine (EDA), employing quantum chemical methods. Saha et al. studied three amine derivatives as the inhibitors of carbon steel corrosion: N1-(2-ami-noethyl)ethane-1,2-diamine (DETA), N1-(2-(2-aminoethylamino)ethyl)ethane-1,2-diamine (TETA) and N1-(2-(2-(2-(2-aminoethylamino)ethylamino)ethyl)ethane-1,2-diamine (PE-HA) by using DFT and MD methods [4]. Furthermore, good correlations were obtained between the results of experimental and theoretical methods in the work of Khadom [32] as well as in the work of Kumar and Kumari [33].

The literature indicates wide applications of amine compounds and their derivatives in corrosion protection. Nevertheless, there are no studies describing how their atomic structure influences the ability to reduce the destructive corrosion process. Therefore, the aim of this work was a quantum-chemical description pertaining to the influence of the chemical structure of amines on their anticorrosion properties. Three structural factors, i.e., the amines subcategory, the length of the hydrocarbon chain (from one to three carbon atoms) attached to the N atom and the form of occurrence of the analyzed amines in an aqueous solution on the ability to form a protector layer by forming coordination bonds and electrostatic interactions were analyzed in this work. An element of novelty of the work is the description of the dependence of the chemical structure and aliphatic amines subcategory on their ability to prevent the corrosion process. 

## 2. Theory and Calculation Method

The quantum chemical calculations reported in this study were carried out at the DFT/B3LYP theory level. Due to consideration of only single molecules in this paper, the dispersion effects were omitted. All calculations were performed at 6-311g-dp basis set using parallel quantum solutions (PQS) suite of ab initio programs and the PQSmol graphical user interface package. 

**Figure 1:**
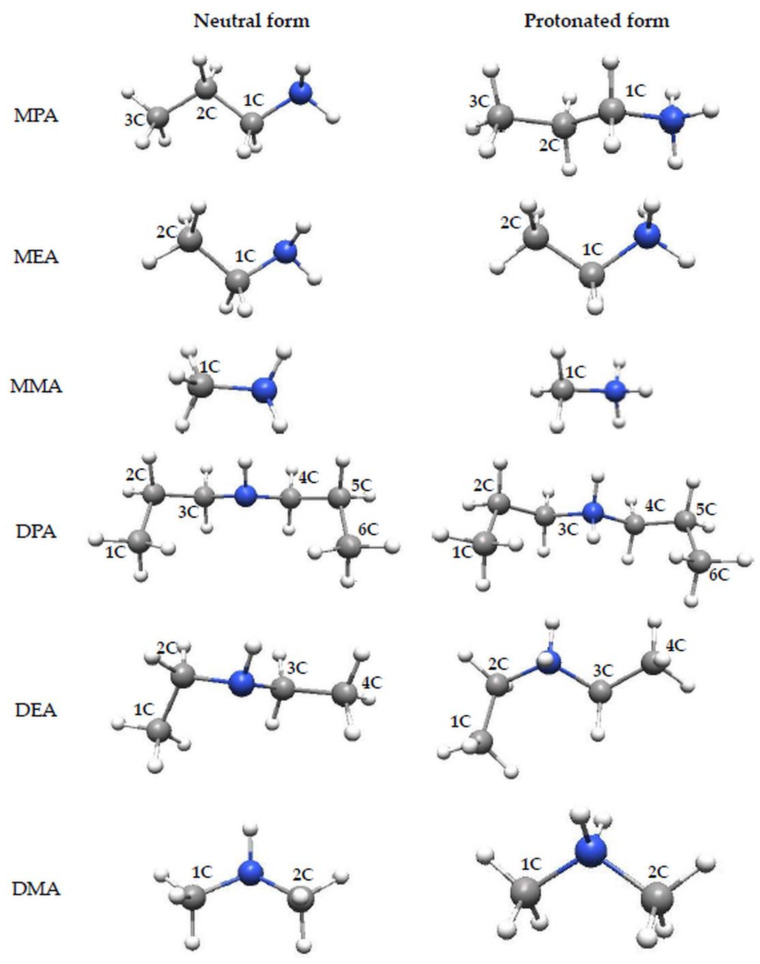

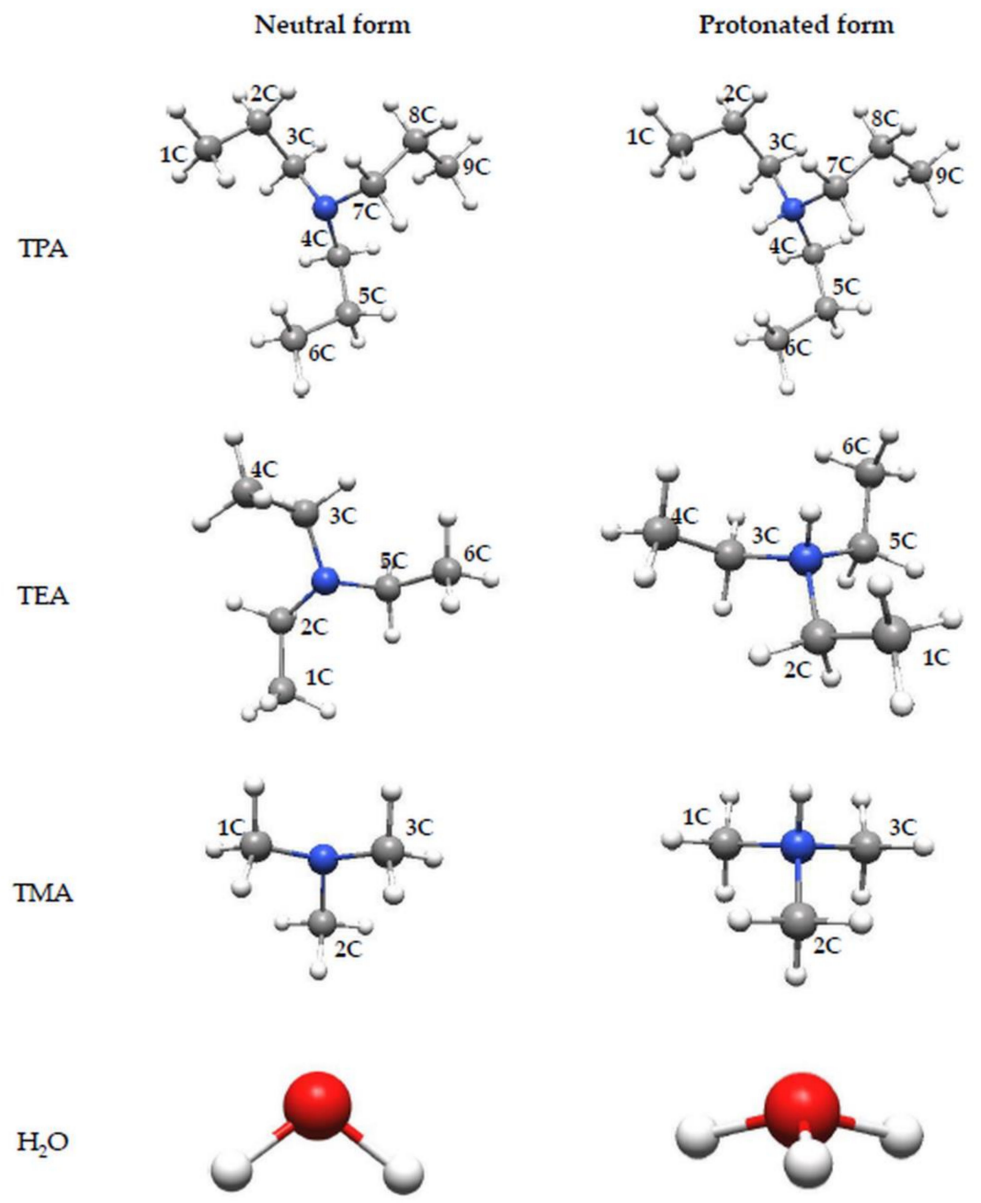
Structures of the analyzed amines and water in neutral and ionized form optimized at the DFT/B3LYP theory level.

This work covers the analysis of anti-corrosion properties of amine compounds differing in chemical structure. In total, nine chemical compounds were analyzed, namely, monopropylamine (MPA), monoethylamine (MEA), monomethylamine (MMA), dipropylamine (DPA), diethylamine (DEA), dimethylamine (DMA), tripropylamine (TPA), triethylamine (TEA) and trimethylamine (TMA). For each considered molecule, the equilibrium geometry shown in Figure 1 was found. In the quantum chemical calculation, the COSMO (conductor-like screening solvation) model was also applied. COSMO is a continuum solvent model, where solute molecules form a cavity within the dielectric continuum of ε permittivity that represent the solvent. In order to obtain closest conditions to the real corrosion system, quantum chemical calculations were performed in the aqueous phase.

The characterization of anti-corrosion properties of the analyzed amines were based on the parameters defined by the highest occupied and the lowest unoccupied molecular orbitals (HOMO and LUMO). The analysis involved consideration of the HOMO–LUMO energy gap (ΔE), electronegativity (χ), dipole moment (µ), global hardness (η), global softness (σ), number of transferred electrons (ΔN) and back-donation energy (ΔE_b-d_) calculated using the following formulas [34,35,36]:

ΔE = E_LUMO_ − E_HOMO_ (1)

(2) $$\chi =\frac{\left(-E_{\mathrm{HOMO}}-E_{\mathrm{LUMO}}\right)}{2}$$

(3) $$\eta =\frac{\left(-E_{\mathrm{HOMO}}+E_{\mathrm{LUMO}}\right)}{2}$$

(4) $$\sigma =\frac{1}{\eta }$$

(5) $$\Delta N=\frac{\chi_{\mathrm{Fe}}-\chi_{\mathrm{inhibitor}}}{2\left(\eta_{\mathrm{Fe}}+\eta_{\mathrm{inhibitor}}\right)}$$

(6) $$\Delta E_{b-d}=-\frac{\eta }{4}$$

The electrostatic interactions of the studied amines with protected metals surfaces were analyzed based on partial charges determined using the Mulliken population analysis.

## 3. Results and Discussion

### 3.1. Molecular Geometry

The optimized geometry of the investigated amine corrosion inhibitors obtained by quantum chemical calculations on the DFT theory level is shown in Figure 1. The obtained structures were considered to be most stable based on three different convergence criteria of geometry optimization procedure, i.e., energy change from previous cycle, maximum allowed gradient component and maximum predicted displacement. Moreover, after the optimization process, the FTIR spectra of all analyzed structures were analyzed. The absence of the imaginary frequencies indicated the correctness of the obtained structures of the analyzed amines in both neutral and protonated forms. Calculations performed on the basis of pKa indicate that the protonated form of the studied amines is mainly present in acidic solutions at pH lower than 10. All geometrical parameters are summarized in Table 1, which shows that the length of the C-N bond does not depend on the amine subcategory (primary, secondary, tertiary) and the length of occurring hydrocarbon chains. For all analyzed organic compounds, a C-N bond length of about 1.47 Å was obtained, which is in agreement with the literature data [37]. The quantum chemical calculations also indicate that the bond length is increased due to the protonation of amine. The comparison of the obtained bond lengths in Table 1 shows that the C-N bond length increased by about 0.06 Å for all analyzed amines as a result of the protonation reaction. This indicates that the degree of bond elongation does not depend on the amine subcategory and occurs only due to the attachment of an additional H atom to the amine group. A lack of changes in the length of the C-C bonds occurring in hydrocarbon chains indicates that the protonation of amines makes structural changes only in the nearest neighborhood of the N atom. On the other hand, the analysis of bond angles shows that all N and C atoms in the analyzed amines have sp^3^ hybridization, and they remain unchanged after the protonation reaction. 

#### Global Molecular Reactivity

In the theoretical analysis of the anticorrosion properties of organic compounds, the frontier molecular orbital (FMO) theory can be successfully used [38]. It allows for the evaluation of their reactivity based on the energy of molecular orbitals, i.e., the highest occupied molecular orbital (HOMO) and the lowest unoccupied molecular orbital (LUMO). The first one is related to the electron donating properties of organic compounds and describes their capacity to donate electrons to the surface of the protected metal. Higher HOMO orbital energy (E_HOMO_) indicates better electron accepting ability of the molecule to the conduction band of the protected metal, which results in greater adsorption properties and consequently leads to higher efficiency of corrosion inhibition [39]. On the other hand, the LUMO orbital energy describes the electron accepting ability of the chemical compound from the conduction band of the protected metal. Thus, the corrosion inhibitors with a lower LUMO orbital energy value will have a higher adsorption capacity on the surface of the protected metal and will inhibit the corrosion process more efficiently [40].

**Figure 2:**
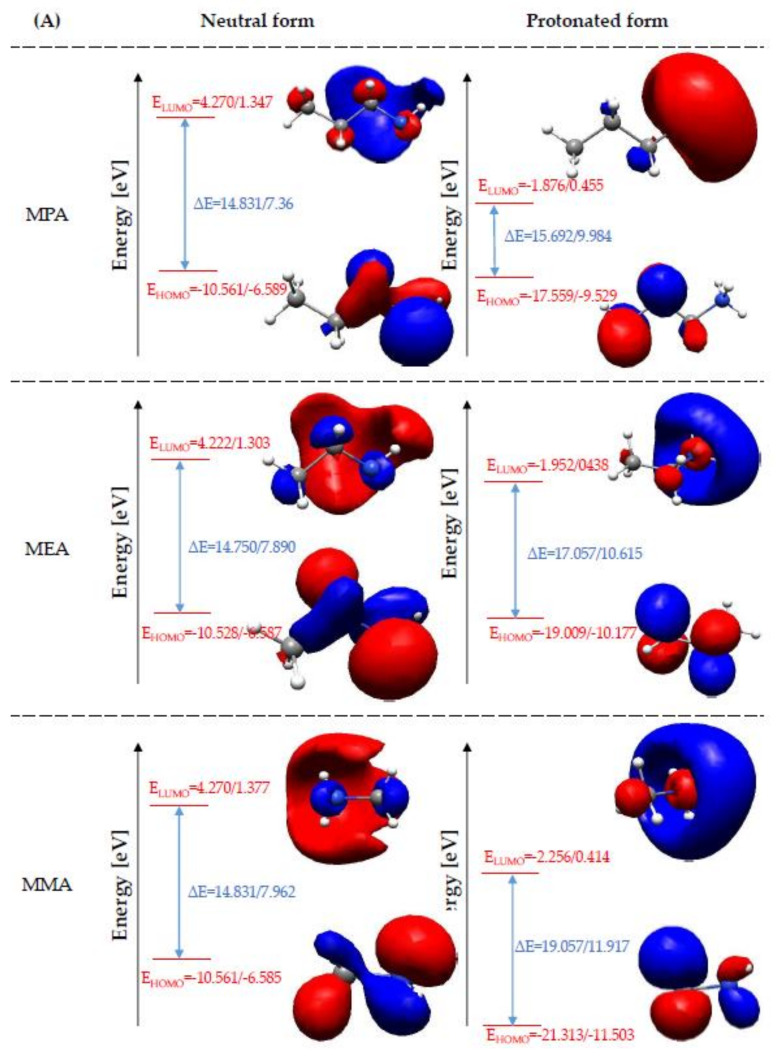

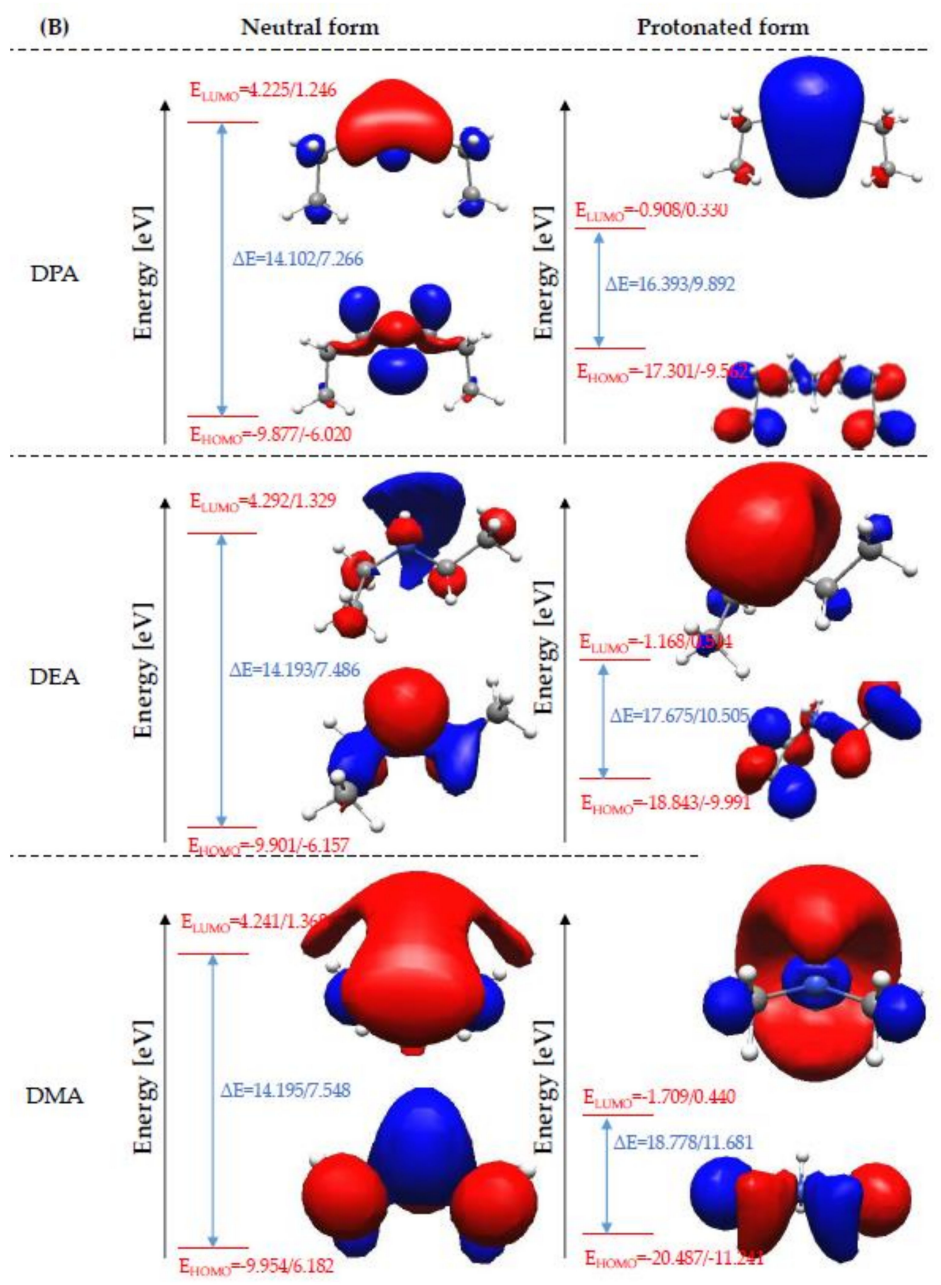

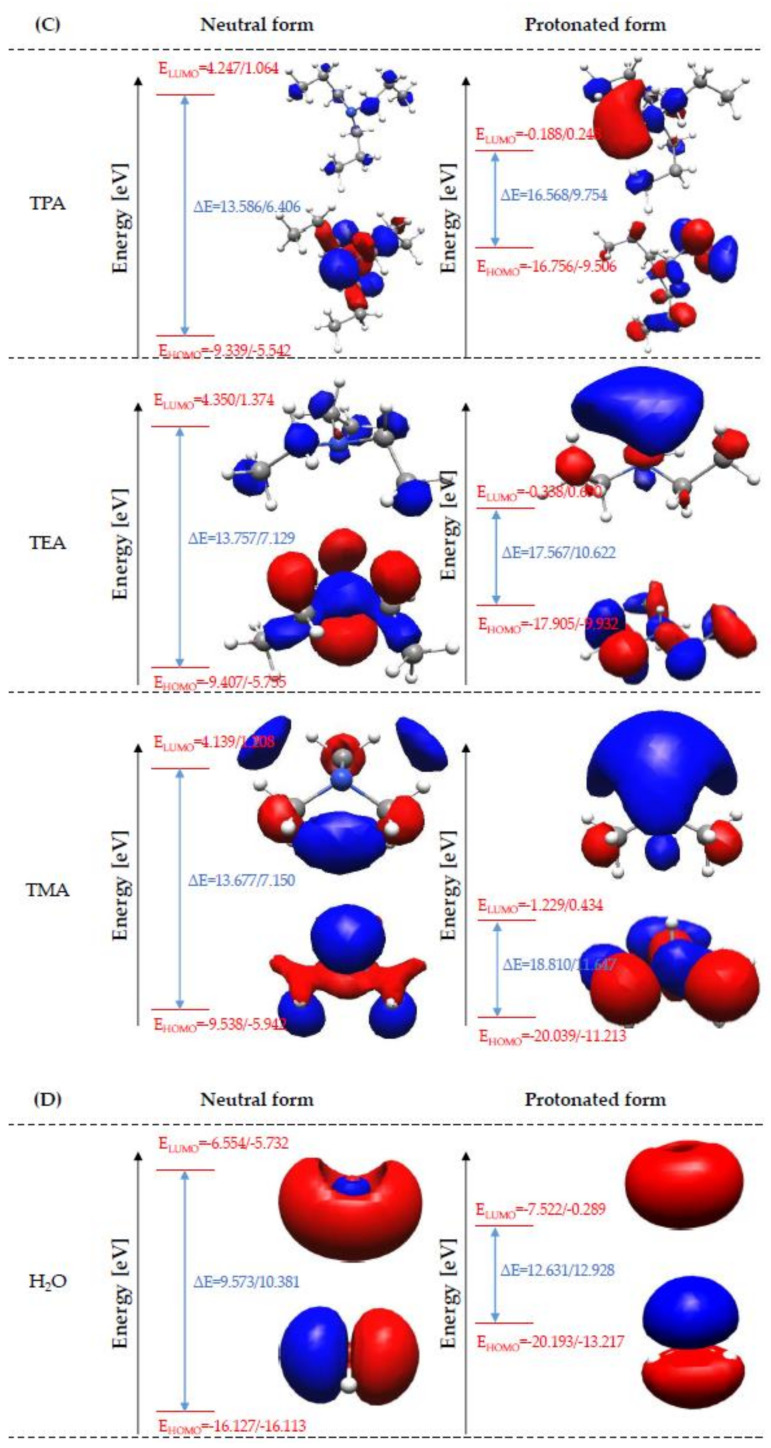
HOMO and LUMO orbital energies (gas phase/water phase) of the primary (**A**), secondary (**B**), tertiary (**C**) amines and (**D**) water in the neutral and ionized form.

Figure 2 represents the E_HOMO_ of the studied amines existing in the neutral and ionized forms. The comparison of E_HOMO_ clearly indicates that the ionized form of the analyzed amines is characterized by much lower values, which means that the protonated form of the studied amines will be characterized by much lower adsorption capacity on the protected metal surface, and, consequently, it will inhibit the corrosion process less efficiently. This is mainly due to the lack of a free electron pair on the nitrogen atom, which was utilized for the H atom attachment and formation of -NH^+^, -NH_2_^+^ or NH_3_^+^ group depending on the amine subcategory. The obtained E_HOMO_ for neutral amines with different hydrocarbon chain lengths are very similar to each other in the primary, secondary and tertiary amine groups. This indicates that the corrosion inhibition efficiency of the neutral amines is not influenced by the number of carbon atoms in hydrocarbon chains but by their number attached to nitrogen atom. A completely opposite dependence was observed for the protonated amines. As it is shown in the Figure 2, in this case, the number of hydrocarbon chains attached to the N atom has lesser influence on the E_HOMO_ than their length. The quantum chemical calculations show that with the increase in the hydrocarbon chain length, the electron donating ability of the primary, secondary and tertiary ionized amines decrease. It is worth noting that this decrease is different for the primary, secondary and tertiary amines. As far as the ionized amines are concerned, the decreases in electron donor properties were 3.754 eV, 3.283 eV and 3.186 eV for the primary, tertiary, secondary amines, respectively. As indicated in Figure 2, quantum chemical calculations taking into account the effect of water as solvent lead to the same conclusions. However, it can be seen that the influence of both the amine order and the hydrocarbon chain length is much weaker for calculations performed in the aqueous phase. The quantum chemical calculations of the corrosion medium molecule indicate that the E_HOMO_ of the water molecule is much lower than that of the tested amines. This indicates a worse ability to adsorb on the surface of the protected metal and the ability to inhibit the corrosion process. As can be seen in Figure 2, the electron accepting ability of amines depends on both the length of the hydrocarbon chain and their subcategory. The comparison of E_LUMO_ values indicates that the protonated amines will better adhere to the metal surface, more efficiently form a protection layer, and, in consequence, inhibit/reduce its corrosion process to a greater extent. As shown in Figure 2, the ability to accept electrons from the conduction band of the metal and consequently the corrosion inhibition efficiency increases among the primary neutral amines in the following order: MEA > MMA and MPA. However, it is worth noting that the difference of the E_LUMO_ value for MMA and MPA is negligible. Thus, it can be concluded that the primary amine with methyl and propyl groups are characterized by a similar electron accepting ability. A slightly different relationship was observed for secondary and tertiary neutral amines. The quantum chemical calculations indicate that the ability to accept the electrons from protected metal of these organic compounds increases as follows: DPA > DMA > DEA for secondary amines and TMA > TPA > TEA for tertiary amines. As it is shown in Figure 2, among the secondary amines, the ethyl substituted compounds are characterized by the worst electron accepting properties for both secondary and tertiary amines. However, the difference between the E_LUMOs_ of TMA and TPA as well as DPA and DMA compounds analyzed in these groups is negligible. The E_LUMO_ obtained for the protonated amines indicate an analogous effect of the hydrocarbon chain length on their electron accepting ability, regardless of the number of substituents. An increase in the hydrocarbon chain length results in an increase in the electron accepting properties of the studied amine corrosion inhibitors. This means that the protonated amines with -CH_3_ substituent will adsorb better on the surface of the protected metal and protect them from the destructive effects of the corrosion process. As regards the influence of the aliphatic amines subcategory on the corrosion inhibition efficiency is concerned, the quantum chemical calculations indicate that the growth of the number of substituents attached to the nitrogen atom causes a decrease in their corrosion inhibition efficiency. Similar to the case of HOMO orbital energy analysis, for LUMO orbital energy, the inclusion of solvent effect in quantum chemical calculations leads to analogous conclusions. On the other hand, E_LUMO_ of neutral and protonated water molecule is lower than E_LUMO_ of the studied amines, which indicates its better adhesive properties to the surface of the protected metal.

**Figure 3:**
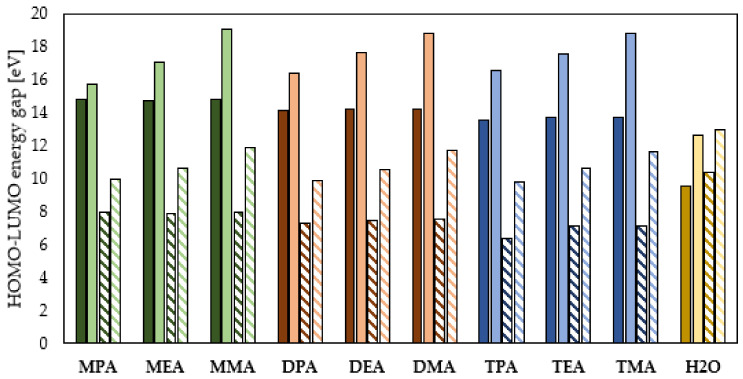
LUMO–HOMO energy gap of the primary (green), secondary (orange), tertiary (blue) amines and water (yellow) in the neutral and ionized form (continuous color: gas phase, pattern-filled column: water phase).

The combined analysis of the E_HOMO_ and E_LUMO_ values allows evaluation of the adsorption of organic compounds and consequently prediction of their corrosion inhibition efficiency. The efficient adsorption process can be suggested by high value of E_HOMO_ and low value of E_LUMO_ [39]. The E_HOMO_ and E_LUMO_ values presented in Figure 2 lead to mutually exclusive conclusions. E_HOMO_ indicates that the corrosion process is better inhibited by the tested aliphatic amines present in the neutral form, while E_LUMO_ indicates that the corrosion process will be more efficiently inhibited by the amines in the ionized form. On the basis of E_HOMO_ and E_LUMO_ alone, it is not possible to clearly indicate which form of amines will be preferred in reducing the destructive effect of the corrosion process. Therefore, further analysis of the investigated aliphatic amines for their kinetic and chemical stability is necessary. A theoretical evaluation of these properties can be performed based on the energy difference of the HOMO orbital and the LUMO orbital. Generally, a lower value of the energy difference of the HOMO and LUMO orbitals indicates higher chemical reactivity and lower kinetic stability of chemical compounds, which results in their higher adsorption efficiency on the surface of the protected metals and consequently a higher degree of corrosion inhibition [4,41]. As shown in Figure 3, the analyzed aliphatic amines in the neutral form are characterized by lower energy difference of the HOMO and LUMO orbitals. Therefore, they will preferably adsorb on the surface of the protected metal, and, consequently, they will inhibit the corrosion process more efficiently. The quantum chemical calculations performed at the DFT/B3LYP theory level indicate that the process inhibition efficiency of the neutral amines increases as follows: primary amines, secondary amines, tertiary amines. That means that an increase in the number of hydrocarbon chains attached to the N atom results in an increase in the process inhibition efficiency of the neutral amines. However, Figure 3 shows that there is no analogous relationship observed for the effect of the hydrocarbon chain length. For the neutral primary amines, it was observed that the highest anti-corrosion properties are characterized by MEA, slightly lower by MPA and the lowest by MMA. It is worth noting that the HOMO–LUMO energy gap difference for the primary amines is minor and amounts to a maximum of 0.081 eV. A slightly higher HOMO–LUMO energy gap of a maximum of 0.093 eV was determined for secondary amines. In this case, the corrosion inhibition efficiency increases as follows: DMA ≈ DEA < DPA. On the other hand, the HOMO–LUMO energy gap analysis of tertiary amines indicates that the corrosion inhibition efficiency increases as follows: TPA > TMA > TEA. Figure 3 clearly indicates that the trends observed for the neutral amines are not applicable to the protonated amines. In this case, no significant influence of the amine subcategory (primary, secondary, tertiary) on their anticorrosion properties was observed. Simultaneously, a stronger effect of the hydrocarbon chain length was observed. The quantum chemical calculations show that the HOMO–LUMO energy gap value decreases with the increase in the hydrocarbon chain length. This indicates that the increase in the hydrocarbon chain length supports the adsorption of the protonated amines on the protected metal surface, leading to more effective protection against the destructive effects of the corrosion process. Similar conclusions were also drawn in the work [8]. As can be seen in Figure 3, the H_2_O molecules present in the system do not change the effect of amine order and hydrocarbon chain length on their chemical and kinetic stability. The comparison of the HOMO–LUMO energy gap determined on the basis of the results obtained in the gas phase and the aqueous phase indicates that the presence of H_2_O molecules enhances the adsorption of neutral and ionized amines on the surface of the protected metal. On the other hand, the lower values of the HOMO–LUMO energy gap obtained for neutral and ionized water molecules indicate that they can adsorb more effectively on the surface of the protected metal and consequently inhibit the corrosion process.

Several researchers link the dipole moment of a corrosion inhibitor with its ability to inhibit the corrosion process [41]. The conducted research has led to the conclusion that a high value of dipole moment favors the accumulation of inhibitor molecules on the surface of the protected metal and its effective protection against corrosion [42]. The values of dipole moments of the both neutral and ionized amines are summarized in Table 2. The comparison of these values clearly confirms the conclusions from the analysis of the energy of the HOMO and LUMO orbitals as well as the energy difference between them. The ionized form of amines will accumulate more efficiently on the surface of the protected metal, protecting it from destructive influence of the corrosion process. The increase in dipole moment of the studied amine molecules depends both on their subcategory (primary, secondary, tertiary) and the hydrocarbon chain length. The highest rise in dipole moment was observed for the primary amines due lack of symmetry of their molecules. The most evident impact of the hydrocarbon chain length on their ability to accumulate on the metal surface was also observed for primary amines. The increase in the dipole moment value decreased with decreasing number of carbon atoms in the hydrocarbon chain. Among the secondary amines, the increase in dipole moment was observed as follows: DMA > DPA > DEA. The lowest dipole moment enhancement of about 0.30 D was observed for the tertiary amines. A clear increase in the dipole moment of the amines occurring in the ionized form indicates that the increase in their corrosion inhibition efficiency is due to the electron cloud deformation and the distribution of partial charges of individual atoms. Therefore, in order to accurately understand and describe the mechanism of corrosion inhibition, it is necessary to analyze the partial charges of individual atoms comprising the studied amines. The comparison of the dipole moments of the analyzed amines determined by quantum chemical calculations in the aqueous phase leads to the exact same conclusions. However, it is worth noting that the presence of water molecules in the corrosion system also causes deformation of the electron cloud, favorably influencing the ability to inhibit the corrosion process of amines occurring in both neutral and protonated form. On the other hand, the lower μ values of neutral and ionized water molecules suggest more evenly distributed electric charge, which indicates that they will adsorb on the surface of the protected metal with lower efficiency.

**Figure 4:**
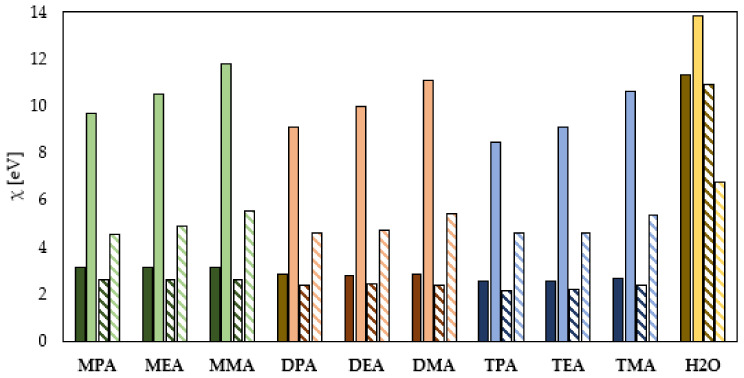
Electronegativity of the primary (green), secondary (orange), tertiary (blue) amines and water (yellow) in the neutral and ionized form (continuous color: gas phase, pattern-filled column: water phase).

Electronegativity is a property that describes the ability of a molecule to accept electrons. As indicated by numerous data in the literature, the chemical compounds characterized by lower electronegativity exhibit stronger anticorrosion properties [4]. Figure 4 shows the quantum chemically determined electronegativity values of the primary, secondary and tertiary amines. The comparison of electronegativity values indicates better efficiency of the corrosion process inhibition by the neutral amines. Simultaneously, the theoretical analysis indicates that for the neutral amines with -NH_2_, -NHR as well as -NR_1_R_2_ groups, the length of hydrocarbon chain has no significant effect on their electronegativity and consequently on their anticorrosion properties. As far as the amines subcategory occurring in the neutral form is concerned, the obtained electronegativity values indicate that the highest efficiency of the corrosion process inhibition will be characteristic for the tertiary amines, the electronegativity value of which is in the range of 2.54 to 2.70 eV. Slightly lower corrosion inhibition abilities are exhibited by the secondary amines with electronegativity of about 2.8 eV, while the lowest anticorrosion properties are exhibited by the primary amines with electronegativity exceeding 3 eV. Other electronegativity dependencies on the chemical structure of the ionized amines were determined. Figure 4 shows that for the primary, secondary and tertiary amines in the ionized form, electronegativity—and consequently their ability to protect metal against corrosion—decreases with increasing hydrocarbon chain length. The highest electronegativity and simultaneously the lowest corrosion inhibition efficiency are characteristic for the amines in the protonated form having only -CH_3_ groups in their structure. Conversely, the lowest value of electronegativity and at the same time the highest efficiency of corrosion inhibition was observed for the amines having the -CH_2_-CH_2_-CH_3_ groups in their structure. At the same time, Figure 4 shows that there is no direct relationship between the order of amines occurring in the protonated form and their ability to inhibit the corrosion process. As indicated in Figure 4, the change in electronegativity values depending on amine order and hydrocarbon chain length is exactly the same in the gas phase and the aqueous phase. However, as in the case of the HOMO–LUMO Energy gap, the electronegativity values obtained from the results obtained by quantum chemical calculations performed in the aqueous phase are much lower, indicating that water molecules can promote the adsorption of amines in both neutral and ionized forms on the surface of the protected metal. As for the water molecule, the quantum chemical calculations lead to interesting conclusions. The values of electronegativity determined on the basis of calculations carried out in the gas phase indicate that a neutral water molecule in both neutral and ionized forms will adsorb worse on the surface of the protected metal. On the other hand, the DFT calculations carried out in the aqueous phase and the electronegativity values determined on their basis indicate that the neutral form of the water molecule will adsorb worse, while the water molecule in the neutral form will adsorb more efficiently on the surface of the protected metal.

**Figure 5:**
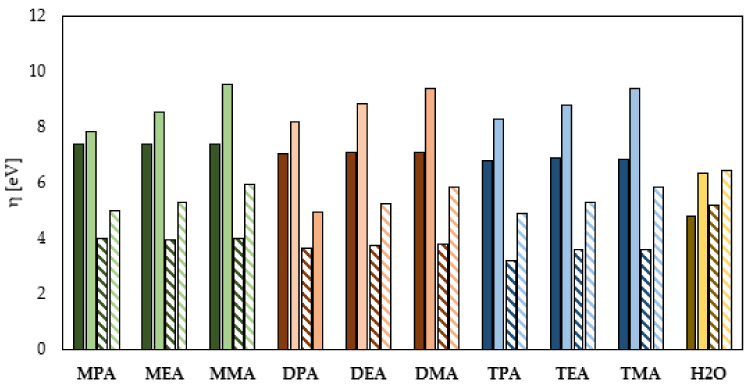
Global hardness of the primary (green), secondary (orange), tertiary (blue) amines and water (yellow) in the neutral and ionized form (continuous color: gas phase, pattern-filled column: water phase).

**Figure 6:**
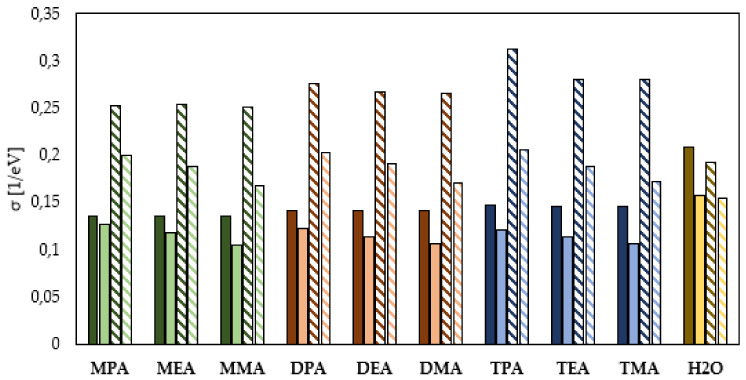
Global softness of the primary (green), secondary (orange), tertiary (blue) amines in the neutral and ionized form (continuous color: gas phase, pattern-filled column: water phase).

The HSAB theory dividing all the compounds into hard and soft particles is a very helpful tool in evaluating the corrosion inhibition properties of organic compounds. Soft chemical compounds with low η (global hardness) and high σ (global softness) are characterized by a higher ability to donate electrons to the metal surface, thus a higher ability to form protective layers and consequently to have higher inhibition potential of the corrosion process. On the other hand, the compounds that belong to the group of hard molecules with high σ value and low η value are characterized by the exactly opposite abilities to inhibit the corrosion process occurring on the surface of metal structures [20]. As shown in Figure 5 and Figure 6, the increase in the neutral amine subcategory causes a decrease in the η value and an increase in the σ value. This means that a decrease in the number of hydrogen atoms in the amine group results in an enhancement of the adhesion of amines to the metal surface, and consequently an increase in the corrosion inhibition efficiency. The quantum chemical calculations also indicate a different influence of the hydrocarbon chain length on the anticorrosive properties of the studied amine compounds. Among the primary amines, having -NH_2_ group in their chemical structure, the highest η value was determined for the amine containing the -CH_3_ group (MMA). Slightly lower η values were determined for the amine involving the -CH_2_-CH_2_-CH_3_ group (MPA) and -CH_2_-CH_3_ group (MEA), respectively. In the case of the neutral secondary amines, the highest comparable η values were determined for DMA and DEA, while the lowest η value was determined for DPA. Among the studied tertiary amines, the value of η determined on the basis of the energy of the HOMO and LUMO orbitals decreased in the following order: TEA > TMA > TPA. The obtained values indicate that in the case of the amines in the neutral form, the ability to adhere on the protected metal surface and consequently its ability to protect against corrosion depend both on their subcategory and hydrocarbon chain length. The analysis of σ values for the studied amine compounds shown in Figure 6 also leads to analogous conclusions. The comparison of the η and σ values presented in Figure 5 and Figure 6 indicates that the protonation of amines changes their anticorrosion properties in a different way. This means that. in general, the protonation of amines leads to an increase in their ability to inhibit the corrosion process, but the increase depends on their order and the length of the hydrocarbon chain. As indicated by the quantum chemical calculations, protonation causes the greatest increase in the corrosion inhibition properties of the tertiary amines. For this group, the increase in the σ value was from about 1.5 eV for TPA to about 2.5 eV for TMA. Slightly lower increases in the σ values were observed for the secondary amines. They ranged from about 1.2 eV for DPA to about 2.3 eV for DMA. The lowest enhancement of surface adhesion to the protected metal was observed for the primary amines, and it ranged from about 0.44 eV for MPA to about 2.1 eV for MMA. The obtained differences also indicate that the increase in the corrosion inhibition efficiency due to amine protonation increases with decreasing hydrocarbon chain length for the primary, secondary and tertiary amines. The quantum chemical calculations performed with the solvent effect taken into account indicate that water molecules present in the corrosion system increase σ while decreasing η value, which indicates that they positively influence the formation of corrosion inhibition potential by the studied molecules. The values of η and σ of the water molecule determined on the basis of quantum chemical calculations performed in the gas phase indicate that the studied amines are characterized by better adsorption capacities on the surface of the protected metal. While the results obtained from quantum chemical calculations performed in the aqueous phase indicate that both the neutral and ionized form of the water molecule will adsorb less efficiently on the metal surface.

### 3.2. Electrostatic Interactions

In order to fully understand the interaction of the corrosion inhibitor with the surface of the protected metal, the electrostatic interactions must be also considered. For this purpose, an analysis of the charges determined with the Mulliken population analysis was carried out. The partial charges of nitrogen atoms, which are mainly responsible for the formation of the protective layer as a result of the electrostatic interactions, are presented in Table 3. The analysis shows that the efficiency of the protection of metals by aliphatic amines due to the electrostatic interactions is mainly determined by their subcategory. As shown in Table 3, the partial charge of the N atom decreases with increasing number of attached hydrocarbon chains. This indicates that the protective layer will be most effectively formed by the primary amines as a result of the electrostatic interactions. Consideration should also be given to the influence of the hydrocarbon chain length. Among the primary amines, a slight decrease in the N atom partial charge was observed. This shows that elongation of the aliphatic chain causes weakening of the electrostatic interactions. In turn, the opposite trend was observed for the secondary and tertiary amines. For these types of organic compounds, the increased hydrocarbon chain length causes a decrease in the partial charge of the N atom, which improves and strengthens the electrostatic interactions of the studied inhibitor molecules with the surface of the protected metal. In order to fully understand the interaction of the corrosion inhibitor molecules with the surface of the protected metal, it is also necessary to analyze the influence of the protonation process of amines on the partial charge of the N atom. Table 3 shows that for the molecules of the investigated aliphatic amines, the attachment of an additional H atom to the N atom as a result of protonation leads to the increase in the partial charge and consequently to the weakening of electrostatic interactions. This increase depends both on the amine subcategory as well as on the length of the aliphatic chain, but the effect of the amine subcategory is definitely stronger. The Mulliken population analysis shows that the primary amines are most susceptible to the weakening of the electrostatic interactions due to their protonation. The addition of an extra H atom increases the partial charge of the N atom by 0.148, 0.134 and 0.128 for MMA, MEA and MPA, respectively. These data indicate that this negative effect is balanced by an increase in the number of C atoms in the aliphatic chains. For the secondary amines, much lower increases in the N atom molecular charge of 0.058, 0.047 and 0.044 were observed for DMA, DEA and DPA, respectively. However, for TMA and TPA, no changes of the N atomic partial charge due to amine protonation were observed; for TEA, the increase was only 0.011. The negative partial charges of the C atoms building aliphatic chains indicate also their participation in the formation of protective layers of metal surfaces as a result of the electrostatic interactions. The data presented in Table 3 show that the partial charge of the C atoms decreases with increasing distance from the N atom. This trend was observed for the primary, secondary and tertiary amines. The effect of protonation of the amine group on the partial charges of the carbon atoms should also be analyzed. Their comparison before and after the protonation reaction indicates that the presence of an additional H atom in the amine group favorably influences the electrostatic interaction of the C atoms with the surface of the protected metal. It is also worth noting that the effect of the protonation reaction on the partial charges of the C atoms is strongest for the primary amines and weakest for the tertiary amines. As far as the influence of the length of the aliphatic chain is concerned, the protonation reaction of the amine group causes the highest improvement of the electrostatic interaction of the atoms directly neighboring with the N atom with the surface of the protected metal. The comparison of partial charges of N atoms shown in Table 3 and Table 4 indicates that electrostatic interactions of the studied neutral amines with the surface of the protected metal are enhanced by the presence of water molecules. In the case of protonated amines the values of partial charges obtained in the aqueous phase are higher than in the gas phase, which in turn indicates the weakening of electrostatic interactions between the metal surface and the nitrogen atom. It is worth noting that the effect of strengthening/weakening electrostatic interactions is directly related to the subcategory of the studied amines and decreases with an increase in the number of hydrocarbon chains attached to the N atom. As far as C atoms are concerned, the quantum chemical investigations indicate that their interactions with the surface of the protected metal are enhanced. This enhancement is much stronger for amines present in neutral form. The quantum chemical calculations carried out both in the gas and aqueous phase indicate stronger interactions of the H_2_O molecule nitrogen atom with the surface of the protected metal. This means that in the actual corrosion system they may adsorb more intensively on the metal blocking the space for the inhibitor molecules leading in consequence to a decrease in its performance.

### 3.3. Number of Transferred Electrons (ΔN) and Back-Donation Energy (ΔEb-d)

Table 5 shows the values of the number of electrons transferred by the inhibitor molecule to the surface of the protected metal. If ΔN > 0, then electrons are transported from the inhibitor molecule to the orbital d of the protected metal. On the other hand, if ΔN < 0, then electrons are transferred from orbital d of the protected metal to the inhibitor molecule [4]. The ΔN values presented in Table 5 indicate that the studied amines accept electrons only in the case of the ionized form occurring in the gas phase. In other cases, electrons are transferred from the inhibitor molecule to the d orbital of the protected metal. For a water molecule, electron transfer to the d orbital of the protected metal occurs only for its protonated form occurring in the aqueous phase. This indicates that the inhibition efficiency of the studied amines improves with an increase in the electron donating capacity of the protected metal. Quantum chemical calculations performed in the gas phase indicate that for neutral amines, with the increase in their order, the E_b-d_ decreases from about 1.85 eV for primary amines to about 1.70 eV for tertiary amines. This means that an increase in the number of hydrocarbon chains attached to the N atom of a neutral amine negatively affects its ability to inhibit the corrosion process. Higher E_b-d_ values have been determined for protonated amines, which means that they possibly have a better ability to inhibit the corrosion process. The application of the solvent model in quantum chemical calculations leads to the exact same conclusions.

## 4. Comparison of DFT Studies with Experimental Findings

The data in the literature include many scientific papers related to the ability of organic compounds to inhibit corrosion. Many of them include both experimental and theoretical works. A comparison of the results obtained by these two routes leads to clear correlations between the parameters obtained by computational methods and the corrosion inhibition performance. For example, three different amine-based compounds, namely, N1-(2-aminoethyl)ethane-1,2-diamine (DETA), N1-(2-(2-aminoethylamino)ethyl)ethane-1,2-diamine (TETA) and N1-(2-(2-(2-aminoethylamino)ethylamino)ethyl)ethane-1,2-diamine (PEHA) differing in the number of -NH- groups in their structure were investigated using potentiodynamic polarization, EIS [43] and DFT methods [4]. The obtained correlations clearly indicate the dependence of the HOMO molecular orbital energy, LUMO molecular orbital energy, the energy difference between them, global softness, electronegativity values and number of transferred electrons on the efficiency of corrosion process inhibition. Similar correlations were obtained for the drug cefixime [42], methionine and tyrosine, as well as their protonated structures [44], 4-(pyridin-3-yl) thiazol-2-amine [21] or triazine-thiourea derivatives [45]. Based on the results and correlations presented in these works, the corrosion inhibition efficiency can be estimated qualitatively directly from quantum chemical calculations.

## 5. Conclusions

The quantum chemical calculations carried out show that the estimation of the anti-corrosion properties is not obvious because the analysis of further parameters leads to different conclusions. The most important of these are:

1. The electron donating ability depends on the form of aliphatic amines in an aqueous solution and their subcategory (primary, secondary, tertiary), but not on the length of the hydrocarbon chains. The efficiency of electron donation to the conduction band of the protected metal is higher for the neutral form of the aliphatic amines and grows with the increase in the number of the hydrocarbon chains attached to the N atom.
2. The protonation of amines leads to an increase in their ability to accept the electrons from the conduction band of the protected metal. Among the studied amines, the highest electron accepting ability is characteristic for the primary amines, and this ability decreases with an increasing number of the hydrocarbon chains attached to the nitrogen atom.
3. The HOMO–LUMO energy gap indicates that the neutral aliphatic amines have lower kinetic stability and higher chemical reactivity and can therefore more efficiently form a protective film on the surface of the metal to be protected.
4. The protonation reaction of the aliphatic amines leads to a deformation of their electron cloud and consequently an improved ability to inhibit the corrosion process. This effect is most evident for the primary amines.
5. The electronegativity values determined from E_HOMO_ and E_LUMO_ and the HSAB theory indicate that the protonation reaction of the aliphatic amines negatively affects their corrosion inhibition performance.
6. The Mulliken population analysis indicates that the electrostatic interactions of the tested corrosion inhibitors occur via the N atoms. The protonation reaction causes a weakening of the electrostatic interactions between the N atom and the surface of the protected metal. The increase in the partial charge of the N atom depends on the amine subcategory and increases as follows: primary amines > secondary amines > tertiary amines.
7. The quantum chemical calculations performed using the COSMO model confirm the conclusions of the gas phase studies regarding the influence of the structure and subcategory of aliphatic amines on their ability to inhibit the corrosion process.

The summary analysis of the parameters determined for a number of aliphatic amines indicates that their protonation changes the character of the formation of coordination bond with the surface of protected metal. For the amines in the neutral form, it is preferred to donate electrons to the metal conduction band, while for the ionized form of amines, it is preferred to accept the electrons from the metal conduction band. However, the aliphatic amines are characterized by higher chemical reactivity, which indicates that this form will be mainly responsible for inhibition of the corrosion process. This is also confirmed by the values of electronegativity as well as global hardness and global softness.

## Figures and Tables

**Table 1:** Bond lengths (Å) and bond angles (°) of the analyzed corrosion inhibitors in the neutral and protonated forms.

Amine	Bond Length (Å)			Bond Angle (°)		
	Gas Phase					
		Neutral Form	Protonated Form		Neutral Form	Protonated Form
MPA	1C-N 2C-1C 3C-2C	1.47 1.53 1.53	1.53 1.52 1.53	2C-1C-N 3C-2C-1C	110.71 113.14	110.90 111.03
MEA	1C-N 2C-1C	1.47 1.53	1.53 1.52	2C-1C-N	110.38	110.74
MMA	1C-N	1.47	1.52	-	-	-
DPA	1C-2C 2C-3C 3C-N N-4C 4C-5C 5C-6C	1.53 1.53 1.47 1.46 1.53 1.53	1.53 1.52 1.52 1.52 1.53 1.53	1C-2C-3C 2C-3C-N 3C-N-4C N-4C-5C 4C-5C-6C	113.89 111.92 113.75 111.92 113.90	114.51 111.79 115.47 111.79 114.51
DEA	1C-2C 2C-N N-3C 3C-4C	1.53 1.47 1.46 1.53	1.52 1.52 1.52 1.52	1C-2C-N 2C-N-3C N-3C-4C	112.38 114.76 110.86	112.51 116.44 110.99
DMA	1C-N N-2C	1.46 1.46	1.51 1.51	1C-N-2C	113.01	114.41
TPA	1C-2C 2C-3C 3C-N N-4C 4C-5C 5C-6C N-7C 7C-8C 8C-9C	1.53 1.54 1.46 1.47 1.54 1.53 1.47 1.55 1.53	1.53 1.53 1.53 1.53 1.53 1.53 1.52 1.52 1.53	1C-2C-3C 2C-3C-N 3C-N-4C 3C-N-7C N-4C-5C 4C-5C-6C N-7C-8C 7C-8C-9C	113.85 113.99 114.32 115.16 114.18 113.90 119.15 116.47	114.55 113.97 114.98 111.49 117.48 117.88 113.73 114.40
TEA	1C-2C 2C-N N-3C 3C-4C N-5C 5C-6C	1.53 1.47 1.47 1.53 1.47 1.53	1.52 1.52 1.52 1.52 1.52 1.52	1C-2C-N 2C-N-3C 2C-N-5C 3C-N-5C N-3C-4C N-5C-6C	113.09 112.11 112.12 112.11 113.09 113.10	112.69 111.94 112.69 111.94 112.70 111.94
TMA	1C-N 2C-N 3C-N	1.46 1.46 1.46	1.51 1.51 1.51	1C-N-2C 2C-N-3C 1C-N-3C	111.64 111.64 111.64	111.80 111.80 111.79
	**Water Phase**					
	**Bond Length (Å)**			**Bond Angle (°)**		
		**Neutral Form**	**Protonated Form**		**Neutral Form**	**Protonated Form**
MPA	1C-N 2C-1C 3C-2C	1.48 1.53 1.53	1.51 1.52 1.53	2C-1C-N 3C-2C-1C	110.73 112.96	110.82 111.26
MEA	1C-N 2C-1C	1.48 1.53	1.51 1.52	2C-1C-N	110.40	110.61
MMA	1C-N	1.48	1.50	-		
DPA	1C-2C 2C-3C 3C-N N-4C 4C-5C 5C-6C	1.53 1.53 1.47 1.47 1.53 1.53	1.53 1.52 1.51 1.51 1.52 1.53	1C-2C-3C 2C-3C-N 3C-N-4C N-4C-5C 4C-5C-6C	114.00 117.79 112.66 111.81 114.00	114.28 111.58 113.01 111.60 114.28
DEA	1C-2C 2C-N N-3C 3C-4C	1.53 1.48 1.48 1.53	1.52 1.52 1.49 1.52	1C-2C-N 2C-N-3C N-3C-4C	112.32 114.39 110.92	112.22 116.28 110.70
DMA	1C-N N-2C	1.47 1.47	1.50 1.50	1C-N-2C	111.71	113.55
TPA	1C-2C 2C-3C 3C-N N-4C 4C-5C 5C-6C N-7C 7C-8C 8C-9C	1.53 1.53 1.47 1.47 1.54 1.53 1.46 1.55 1.53	1.53 1.53 1.53 1.53 1.54 1.53 1.51 1.53 1.53	1C-2C-3C 2C-3C-N 3C-N-4C 3C-N-7C N-4C-5C 4C-5C-6C N-7C-8C 7C-8C-9C	113.75 113.82 112.99 112.99 114.20 113.89 119.46 116.48	114.22 114.09 114.36 115.61 113.99 114.03 116.91 116.71
TEA	1C-2C 2C-N N-3C 3C-4C N-5C 5C-6C	1.53 1.47 1.48 1.53 1.46 1.53	1.52 1.52 1.51 1.52 1.52 1.52	1C-2C-N 2C-N-3C 2C-N-5C 3C-N-5C N-3C-4C N-5C-6C	112.92 111.24 112.18 112.06 113.01 113.06	112.93 111.85 111.11 112.00 112.60 112.45
TMA	1C-N 2C-N 3C-N	1.47 1.47 1.47	1.50 1.50 1.50	1C-N-2C 2C-N-3C 1C-N-3C	110.49 111.35 111.36	111.63 111.63 111.63

**Table 2:** Dipole moment (µ) of the neutral and protonated amines.

Amine	µ (Debye)			
	Gas Phase		Water Phase	
	Neutral Form	Protonated Form	Neutral Form	Protonated Form
MPA	1.34	6.74	1.77	8.15
MEA	1.41	4.14	1.83	5.12
MMA	1.46	2.34	1.82	2.96
DPA	1.04	1.90	1.43	2.40
DEA	1.02	1.97	1.41	2.69
DMA	1.10	1.70	1.39	2.26
TPA	0.69	0.99	0.78	1.44
TEA	0.69	0.41	0.89	1.49
TMA	0.69	1.03	0.81	1.43

**Table 3:** Atomic charges determined using the Mulliken population analysis.

		Atom	Neutral Amine	Ionized Amine	Atom	Neutral Amine	Ionized Amine
Primary amine	MMA	N	−0.472	−0.324	1C	−0.205	−0.210
	MEA	N	−0.457	−0.323	1C 2C	−0.119 −0.297	−0.162 −0.292
	MPA	N	−0.457	−0.329	1C 2C 3C	−0.095 −0.227 −0.299	−0.131 −0.253 −0.292
Secondary amine	DMA	N	−0.385	−0.327	1C 2C	−0.193 −0.193	−0.197 −0.197
	DEA	N	−0.379	−0.332	1C 2C 3C 4C	−0.279 −0.193 −0.193 −0.302	−0.303 −0.142 −0.141 −0.302
	DPA	N	−0.383	−0.339	1C 2C 3C 4C 5C 6C	−0.283 −0.241 −0.080 −0.080 −0.241 −0.283	−0.316 −0.252 −0.112 −0.112 −0.252 −0.317
Tertiary amine	TMA	N	−0.331	−0.333	1C 2C 3C	−0.182 −0.182 −0.182	−0.190 −0.190 −0.190
	TEA	N	−0.353	−0.342	1C 2C 3C 4C 5C 6C	−0.266 −0.113 −0.113 −0.226 −0.113 −0.226	−0.298 −0.138 −0.138 −0.298 −0.138 −0.298
	TPA	N	−0.368	−0.368	1C 2C 3C 4C 5C 6C 7C 8C 9C	−0.280 −0.238 −0.081 −0.074 −0.224 −0.289 −0.100 −0.224 −0.295	−0.289 −0.282 −0.101 −0.100 −0.282 −0.289 −0.101 −0.282 −0.289
H_2_O		O	−0.474	−0.208	-	-	-

**Table 4:** Atomic charges determined using Mulliken population analysis in water phase.

		Atom	Neutral Amine	Ionized Amine	Atom	Neutral Amine	Ionized Amine
Primary amine	MMA	N	−0.545	−0.288	1C	−0.218	−0.204
	MEA	N	−0.528	−0.292	1C 2C	−0.119 −0.316	−0.134 −0.313
	MPA	N	−0.530	−0.298	1C 2C 3C	−0.010 −0.232 −0.320	−0.106 −0.242 −0.316
Secondary amine	DMA	N	−0.443	−0.297	1C 2C	−0.209 −0.209	−0.194 −0.194
	DEA	N	−0.430	−0.319	1C 2C 3C 4C	−0.318 −0.103 −0.121 −0.300	−0.317 −0.115 −0.120 −0.316
	DPA	N	−0.409	−0.331	1C 2C 3C 4C 5C 6C	−0.307 −0.246 −0.087 −0.087 −0.246 −0.307	−0.323 −0.251 −0.094 −0.094 −0.251 −0.323
Tertiary amine	TMA	N	−0.368	−0.318	1C 2C 3C	−0.203 −0.203 −0.203	−0.189 −0.189 −0.189
	TEA	N	−0.370	−0.350	1C 2C 3C 4C 5C 6C	−0.288 −0.117 −0.117 −0.288 −0.117 −0.288	−0.307 −0.124 −0.124 −0.307 −0.124 −0.307
	TPA	N	−0.380	−0.362	1C 2C 3C 4C 5C 6C 7C 8C 9C	−0.314 −0.239 −0.111 −0.077 −0.243 −0.300 −0.088 −0.228 −0.302	−0.330 −0.250 −0.108 −0.134 −0.255 −0.324 −0.108 −0.228 −0.302
H_2_O		O	−0.570	−0.215	-	-	-

**Table 5:** Number of transferred electrons (ΔN) and back-donation energy (ΔE_b-d_) of aliphatic amines and water.

Amine	ΔN				E_b-d_ (eV)			
	Gas Phase		Water Phase		Gas Phase		Water Phase	
	Neutral Form	Protonated Form	Neutral Form	Protonated Form	Neutral Form	Protonated Form	Neutral Form	Protonated Form
MPA	0.32	−0.12	0.67	0.34	1.85	1.96	0.99	1.25
MEA	0.32	−0.12	0.67	0.29	1.84	2.13	0.98	1.32
MMA	0.32	−0.20	0.67	0.20	1.85	2.38	0.99	1.48
DPA	0.36	−0.07	0.76	0.33	1.76	2.04	0.90	1.23
DEA	0.36	−0.12	0.73	0.30	1.77	2.20	0.94	1.31
DMA	0.36	−0.17	0.73	0.21	1.77	2.34	0.94	1.46
TPA	0.39	−0.03	0.90	0.34	1.69	2.07	0.80	1.21
TEA	0.39	−0.07	0.80	0.31	1.71	2.20	0.89	1.32
TMA	0.38	−0.15	0.77	0.22	1.70	2.35	0.89	1.46
H_2_O	−0.36	−0.47	−0.29	0.09	1.20	1.58	1.30	1.61

## Data Availability

Data is contained within the article.

## References

[1] Hamadi L., Mansouri S., Oulmi K., Kareche A. (2018). The use of amino acids as corrosion inhibitors for metals: A review. Egypt. J. Pet..

[2] Finšgar M., Jackson J. (2014). Application of corrosion inhibitors for steels in acidic media for the oil and gas industry: A review. Corros. Sci..

[3] Zhang H.L., Ma T.F., Gao L.X., Zhang D.Q., Wei G.A., Yan H.B., Wei S.L. (2020). Vapor phase assembly of urea–amine compounds and their protection against the atmospheric corrosion of carbon steel. J. Coat. Technol. Res..

[4] Saha S.K., Murmu M., Murmu N.C., Obot I.B., Banerjee P. (2018). Molecular level insights for the corrosion inhibition effectiveness of three amine derivatives on the carbon steel surface in the adverse medium: A combined density functional theory and molecular dynamics simulation study. Surf. Interfaces.

[5] Ali A.A.I., El-dougdoug W.I.A. (2017). Green Chemistry Letters and Reviews Preparation and evaluation of amido poly amine surfactant based on Melia azedarach seeds oil as corrosion inhibitor of C-steel in 2.0 M HCl pickling medium. Green Chem. Lett. Rev..

[6] Dwivedi D., Lepková K., Becker T. (2017). Carbon steel corrosion: A review of key surface properties and characterization methods. RSC Adv..

[7] Guo L., Zhu S., Zhang S., He Q., Li W. (2014). Theoretical studies of three triazole derivatives as corrosion inhibitors for mild steel in acidic medium. Corros. Sci..

[8] Topal E., Gece G. (2017). Untangling the inhibition effects of aliphatic amines on silver corrosion: A computational study. Chem. J. Mold..

[9] Chauhan D.S., Quraishi M.A., Jafar Mazumder M.A., Ali S.A., Aljeaban N.A., Alharbi B.G. (2020). Design and synthesis of a novel corrosion inhibitor embedded with quaternary ammonium, amide and amine motifs for protection of carbon steel in 1 M HCl. J. Mol. Liq..

[10] Rihan R., Shawabkeh R., Al-Bakr N. (2014). The effect of two amine-based corrosion inhibitors in improving the corrosion resistance of carbon steel in sea water. J. Mater. Eng. Perform..

[11] Lai Y., Gao Y., Jin Y., Wen L. (2021). Study of methionine as green corrosion inhibitor for TWIP steel in neutral chloride solution Study of methionine as green corrosion inhibitor for TWIP steel in neutral chloride solution. Mater. Res. Express.

[12] Amin M.A., Khaled K.F., Mohsen Q., Arida H.A. (2010). A study of the inhibition of iron corrosion in HCl solutions by some amino acids. Corros. Sci..

[13] Wang T., Wang J., Wu Y. (2015). The inhibition effect and mechanism of l-cysteine on the corrosion of bronze covered with a CuCl patina. Corros. Sci..

[14] Wang D., Gao L., Zhang D., Yang D., Wang H., Lin T. (2016). Experimental and theoretical investigation on corrosion inhibition of AA5052 aluminium alloy by l-cysteine in alkaline solution. Mater. Chem. Phys..

[15] Zhang Z., Tian N., Zhang W., Huang X., Ruan L., Wu L. (2016). Inhibition of carbon steel corrosion in phase-change-materials solution by methionine and proline. Corros. Sci..

[16] Nady H. (2017). Tricine [N-(Tri(hydroxymethyl)methyl)glycine]—A novel green inhibitor for the corrosion inhibition of zinc in neutral aerated sodium chloride solution. Egypt. J. Pet..

[17] Amin M.A., Khaled K.F. (2010). Copper corrosion inhibition in O_2_-saturated H_2_SO_4_ solutions. Corros. Sci..

[18] El Ibrahimi B., Bazzi L., El Issami S. (2020). The role of pH in corrosion inhibition of tin using the proline amino acid: Theoretical and experimental investigations. RSC Adv..

[19] Caldona E.B., Wipf D.O., Smith D.W. (2021). Characterization of a tetrafunctional epoxy-amine coating for corrosion protection of mild steel. Prog. Org. Coat..

[20] Malinowski S., Jaroszyńska-Wolińska J., Herbert T. (2018). Theoretical predictions of anti-corrosive properties of THAM and its derivatives. J. Mol. Model..

[21] Farahati R., Behzadi H., Mousavi-Khoshdel S.M., Ghaffarinejad A. (2020). Evaluation of corrosion inhibition of 4-(pyridin-3-yl) thiazol-2-amine for copper in HCl by experimental and theoretical studies. J. Mol. Struct..

[22] Sığırcık G., Yildirim D., Tüken T. (2017). Synthesis and inhibitory effect of N,N’-bis(1-phenylethanol)ethylenediamine against steel corrosion in HCl Media. Corros. Sci..

[23] Shahraki M., Dehdab M., Elmi S. (2016). Theoretical studies on the corrosion inhibition performance of three amine derivatives on carbon steel: Molecular dynamics simulation and density functional theory approaches. J. Taiwan Inst. Chem. Eng..

[24] Asegbeloyin J.N., Ejikeme P.M., Olasunkanmi L.O., Adekunle A.S., Ebenso E.E. (2015). A novel schiffbase of 3-acetyl-4-hydroxy-6-methyl-(2H)pyran- 2-one and 2,2′-(ethylenedioxy)diethylamine as potential corrosion inhibitor for mild steel in acidic medium. Materials.

[25] Al-Sabagh A.M., Nasser N.M., Farag A.A., Migahed M.A., Eissa A.M.F., Mahmoud T. (2013). Structure effect of some amine derivatives on corrosion inhibition efficiency for carbon steel in acidic media using electrochemical and Quantum Theory Methods. Egypt. J. Pet..

[26] Li X., Pearson P., Yang Q., Puxty G., Feron P., Xiao D. (2020). A study of designer amine 4-amino-1-propyl-piperidine against the corrosion of carbon steel for application in CO2 capture. Int. J. Greenh. Gas Control.

[27] Campbell K.L.S., Zhao Y., Hall J.J., Williams D.R. (2016). The effect of CO2-loaded amine solvents on the corrosion of a carbon steel stripper. Int. J. Greenh. Gas Control.

[28] Iroha N.B., Dueke-Eze C.U., James A.O., Fasina T.M. (2021). Newly synthesized N-(5-nitro-2-hydroxybenzylidene)pyridine-4-amine as a high-potential inhibitor for pipeline steel corrosion in hydrochloric acid medium. Egypt. J. Pet..

[29] Yadav M., Kumar S., Sharma U., Yadav P.N. (2013). Substituted amines as corrosion inhibitors for N80 steel in 15% HCl. J. Mater. Environ. Sci..

[30] Boughoues Y., Benamira M., Messaadia L., Bouider N., Abdelaziz S. (2020). Experimental and theoretical investigations of four amine derivatives as effective corrosion inhibitors for mild steel in HCl medium. RSC Adv..

[31] Shihab M.S., Al-Doori H.H. (2014). Experimental and theoretical study of [N-substituted] p-aminoazobenzene derivatives as corrosion inhibitors for mild steel in sulfuric acid solution. J. Mol. Struct..

[32] Khadom A.A. (2017). Quantum chemical calculations of some amines corrosion inhibitors/ copper alloy interaction in hydrochloric acid. J. Mater. Environ. Sci..

[33] Kumar H., Kumari M. (2021). Experimental and theoretical investigation of 3,3′-diamino dipropyl amine: Highly efficient corrosion inhibitor for carbon steel in 2 N HCl at normal and elevated temperatures. J. Mol. Struct..

[34] Tuzun B., Bhawsar J. (2021). Quantum chemical study of thiaozole derivatives as corrosion inhibitors based on density functional theory. Arab. J. Chem..

[35] Sait N., Aliouane N., Toukal L., Hammache H., Al-Noaimi M., Helesbeux J.J., Duval O. (2021). Synthesis of ethylene bis [(2-hydroxy-5,1,3-phenylene) bis methylene] tetraphosphonic acid and their anticorrosive effect on carbon steel in 3% NaCl solution. J. Mol. Liq..

[36] Khnifira M., Mahsoune A., Belghiti M.E., Khamar L., Sadiq M., Abdennouri M., Barka N. (2020). HF and SiF4 adsorption on carbon graphite (1 1 1) surface in aqueous medium: A combined DFT and MD simulation approach. Mater. Today Proc..

[37] Wichmann K.A., Boyd P.D.W., Söhnel T., Allen G.R., Phillips A.R.J., Cooper G.J.S. (2007). Characterization of dicarboxylic salts of protonated triethylenetetramine useful for the treatment of copper-related pathologies. Cryst. Growth Des..

[38] Zheng M., Li Y., Ding K., Zhang Y., Chen W., Lin W. (2020). A boron-decorated melon-based carbon nitride as a metal-free photocatalyst for N2 fixation: A DFT study. Phys. Chem. Chem. Phys..

[39] El Adnani Z., Mcharfi M., Sfaira M., Benzakour M., Benjelloun A.T., Ebn Touhami M., Hammouti B., Taleb M. (2012). DFT study of 7-R-3methylquinoxalin-2(1H)-ones (R=H; CH 3; Cl) as corrosion inhibitors in hydrochloric acid. Int. J. Electrochem. Sci..

[40] Khadiri A., Saddik R., Bekkouche K., Aouniti A., Hammouti B., Benchat N., Bouachrine M., Solmaz R. (2016). Gravimetric, electrochemical and quantum chemical studies of some pyridazine derivatives as corrosion inhibitors for mild steel in 1 M HCl solution. J. Taiwan Inst. Chem. Eng..

[41] Wazzan N.A. (2015). DFT calculations of thiosemicarbazide, arylisothiocynates, and 1-aryl-2,5-dithiohydrazodicarbonamides as corrosion inhibitors of copper in an aqueous chloride solution. J. Ind. Eng. Chem..

[42] Diki N.Y.S., Gbassi G.K., Ouedraogo A., Berte M., Trokourey A. (2018). Aluminum corrosion inhibition by cefixime drug: Experimental and DFT studies. J. Electrochem. Sci. Eng..

[43] Migahed M.A., Attia A.A., Habib R.E. (2015). Study on the efficiency of some amine derivatives as corrosion and scale inhibitors in cooling water systems. RSC Adv..

[44] Zor S., Kandemirli F., Bingul M. (2009). Inhibition effects of methionine and tyrosine on corrosion of iron in HCl solution: Electrochemical, FTIR, and quantum-chemical study. Prot. Met. Phys. Chem. Surf..

[45] Paul P.K., Yadav M. (2020). Investigation on corrosion inhibition and adsorption mechanism of triazine-thiourea derivatives at mild steel / HCl solution interface: Electrochemical, XPS, DFT and Monte Carlo simulation approach. J. Electroanal. Chem..

